# Physicochemical, Textural, and Rheological Properties of Bigels Prepared from Large Yellow Croaker Myofibrillar Proteins and κ-Carrageenan

**DOI:** 10.3390/foods15173017

**Published:** 2026-08-27

**Authors:** Zhongyang Ren, Tengteng Yang, Qiaochu Li, Zhanming Li, Caili Fu

**Affiliations:** 1 College of Ocean Food and Biological Engineering, Jimei University, Xiamen 361021, China; rzy0529@jmu.edu.cn (Z.R.); 17698583037@163.com (T.Y.); 2 National University of Singapore (Suzhou) Research Institute, Suzhou Industrial Park, Suzhou 215123, China; judy_li5123@163.com; 3 School of Grain Science and Technology, Jiangsu University of Science and Technology, Zhenjiang 212100, China; lizhanming@just.edu.cn

**Keywords:** large yellow croaker, myofibrillar proteins, κ-carrageenan, bigel, rheological properties

## Abstract

Bigels combine the advantages of hydrogel and oleogel matrices, enabling simultaneous encapsulation of both hydrophilic and lipophilic compounds with good ductility and water retention. Bigels were prepared from large yellow croaker myofibrillar protein (MP)–soybean oil oleogels and κ-carrageenan hydrogels. All the bigels exhibited a milky white, solid-like self-supporting structure. Increasing the oleogel fraction enhanced droplet interactions and uniformity. Hardness peaked at 11.67 g at a 5:5 (*w*/*w*) oleogel-to-hydrogel ratio. The rheological tests revealed shear-thinning behavior across all samples, with apparent viscosity increasing progressively with oleogel content. The highest thixotropic recovery (71.07%) was observed at an oleogel-to-hydrogel ratio of 6:4 (*w*/*w*). All bigels underwent structural destabilization above 40 °C and complete liquefaction above 60 °C, indicating thermal reversibility. This study elucidates how varying the oleogel-to-hydrogel ratio modulates the physicochemical properties of bigels, providing a theoretical basis for developing high-value products from large yellow croaker.

## 1. Introduction

Gels are three-dimensional colloidal networks generated through the association and cross-linking of polymeric chains or dispersed particles under suitable environmental conditions [1]. Due to their distinctive structural organization and functional versatility, gel-based systems have gained extensive applications in food manufacturing. In food processing, gels are frequently used as key components in the production of jelly, candy, yogurt, and other related products. Based on the nature of the continuous phase, gel systems are generally categorized into hydrogels and oleogels [2]. As a polymeric material with a three-dimensional network structure, hydrogels have drawn widespread research interest owing to their exclusive physicochemical traits and promising application potential [3]. Hydrogels possess high water content while maintaining desirable properties including excellent biocompatibility and stretchability, yet they fail to adequately mimic food textures and are difficult to apply directly in food systems [4]. Oleogels can form three-dimensional network structures via crystalline particles but exhibit poor loading and delivery capacity for hydrophilic components despite their favorable structural stability [5]. The inherent shortcomings associated with individual gel systems have stimulated increasing interest toward constructing biphasic gel structures that combine the superior properties of every single phase.

Bigels integrate the outstanding properties derived from hydrogel and oleogel matrices to construct dual-phase frameworks by shear mixing of the two components at different ratios and certain temperatures, thereby enabling simultaneous delivery of hydrophilic and lipophilic substances with favorable moisture retention and ductility [1]. Based on the spatial distribution of the two constituent phases, bigels are routinely categorized into three types: hydrogel-entrapped oleogel systems (O/W), oleogel-entrapped hydrogel systems (W/O), and bicontinuous systems [6]. Bigels exhibit superior stability due to their unique structure. Hydrogels and oleogels allow the mobile phase to form a super-dispersion within the 3D network of the gel for inhibiting aggregation and flocculation [7]. Prior studies have shown that adjusting the composition and ratio of bigels tailors their structure, rheology, and chewiness, offering potential for dysphagia-oriented food design. Embedding β-carotene in bigels could improve their photostability and thermal tolerance, while the amount of β-carotene released increased in simulated intestinal fluid, with the maximum release observed at an oleogel content of 75% [8]. Beef burgers with the bigel addition of 50% could reduce fat as much as possible without changing their textural properties [9]. Compared with single gels, the hydrogel and oleogel components within bigels exhibit cooperative interactions, contributing to enhanced structural stability [10]. Therefore, investigating the influences of composite gelators on the physicochemical characteristics of bigels is essential.

Over recent years, functional food applications have seen a marked rise in the use of gels fabricated from naturally occurring polymers, including proteins and polysaccharides, owing to their edibility, biocompatibility, biodegradability, and economic viability [11]. For example, bigels engineered from tea polysaccharide-egg white protein nanoparticles have been found to display superior interfacial viscoelasticity and a hierarchically structured three-dimensional gel framework, based on assessments of their oil-water (O/W) interfacial dynamics [12]. MPs are salt-soluble amphiphilic proteins with excellent functional properties including emulsification, gelation, and water retention [13]. Large yellow croaker, as a marine biological resource, is rich in MPs, which constitute 55–60% of fish protein, with excellent emulsifying and gelling properties [14]. κ-Carrageenan is widely utilized as a stabilizing and thickening agent within food manufacturing systems [15]. In previous studies, myofibrillar proteins and κ-carrageenan have been used in the construction of bigel systems. Zhang et al. [10] prepared bigels using pork MP hydrogels and beeswax oleogels as raw materials, demonstrating that myofibrillar protein can elevate the mechanical properties and rheological robustness of bigels. Zampouni et al. [16] constructed oleogel-embedded hydrogel (O/W) bigels using gelatin/κ-carrageenan as the aqueous phase, indicating that gelatin and κ-carrageenan can endow bigels with higher hardness and thermal stability. Furthermore, κ-carrageenan can enhance the thermal stability of oleogels, solving the problem of poor thermal durability in single-component oleogels [17]. However, studies on the construction of bigels using marine fish MPs as oleogelators remain limited at present. In addition, the synergistic mechanism between MPs and κ-carrageenan, as well as the regulatory effects of their interaction on the physical and chemical performances of bigels, have not yet been clarified.

To date, few studies have focused on marine fish MPs as oleogel-forming matrices, while the synergistic interactions between MPs and κ-carrageenan hydrogel still lack systematic clarification. Herein, bigels were constructed using large yellow croaker MPs and κ-carrageenan as oleogel and hydrogel phases, respectively. The modulatory rules of oleogel-hydrogel volume ratios on microstructural features, textural traits, and viscoelastic performances were comprehensively investigated to uncover the influences of phase fractions on micromorphology, non-covalent molecular interactions, and rheological responses of marine protein-based bigels. The acquired results may compensate for the existing research deficit of marine MP biphasic gels and may provide reliable theoretical references for high-value reutilization of large yellow croaker processing wastes and the exploitation of natural fat replacers in functional food fabrication.

## 2. Materials and Methods

### 2.1. Materials

Fresh large yellow croaker was purchased from Xinhua Du Shopping Mall Co., Ltd. (Xiamen, China), which was slaughtered by the market from which they were purchased. κ-Carrageenan was purchased from Sigma-Aldrich Co., Ltd. (Shanghai, China). Soybean oil was purchased from Yihai Kerry Arawana Holdings Co., Ltd. (Shanghai, China). Glycerol and silicone oil were purchased from Xilong Scientific Co., Ltd. (Yichang, China). Nile red and fluorescein isothiocyanate (FITC) were purchased from Aladdin Co., Ltd. (Shanghai, China). Fluorescent Brightener 28 was purchased from Mallcom Technology Co., Ltd. (Shanghai, China).

### 2.2. Materials Extraction of MP

MPs were isolated in accordance with the reported protocol with slight modifications [18]. The head, tail, skin, internal organs, and red meat of large yellow croaker were thoroughly excised. The fresh dorsal white muscle after pretreatment was immersed in phosphate buffer with low ionic strength (0.02 mol/L Na_2_HPO_4_, 0.02 mol/L NaH_2_PO_4_, 0.05 mol/L NaCl, pH 7) and homogenized with a food processor (MQ-100, Bolang, Shanghai, China). Crushed fish meat was placed at 4 °C for 15 min and centrifuged at 4 °C and 1400× *g* for 10 min using a centrifuge (W21R, Hukang, Changsha China). The sediment was homogenized with 4 volumes of carbonate buffer (0.02 mol/L Na_2_HPO_4_, 0.02 mol/L NaH_2_PO_4_, 0.45 mol/L NaCl, pH 7). Post-homogenization, the mixture was stored at 4 °C overnight, followed by centrifugation at 12,500× *g* for 10 min at the same temperature. The recovered supernatant was diluted 10-fold with ice-cold pure water and centrifuged at 4800 r/min for 10 min at 4 °C to collect MPs.

### 2.3. Preparation of Oleogels

Oleogels were prepared according to previously reported methods, with slight modifications [19]. MPs were dispersed in deionized water to prepare a 2% (*w*/*v*) suspension and stirred with a magnetic stirrer (DF-101Z, Lichen Bangxi Instrument, Shanghai, China) for 4 h to obtain a stable protein suspension. MP suspension was heated and stirred at 80 °C for 30 min in a water bath magnetic stirrer (CJJ78-1, Meixiang Instruments, Shanghai, China), followed by cooling down to 25 °C. The homogenization of the MP suspension was carried out at 8000× *g* for 2 min using a high-speed homogenizer (FA25, FLUKO, Shanghai, China). MP suspension was mixed with soybean oil at an oil-water ratio of 8:2 (*v*/*v*), followed by the addition of glycerol equal in volume to the MP suspension. The mixture was homogenized at 22,000× *g* for 2 min to form an emulsion, and dried at 60 °C for 7 h to obtain oleogels.

### 2.4. Preparation of κ-Carrageenan Hydrogels

Hydrogels based on κ-carrageenan were fabricated according to an earlier reported method [20]. κ-carrageenan was dissolved in deionized water to obtain a 1% (*w*/*v*) solution, which was heated with stirring in an 80 °C water bath until complete dissolution and subsequently cooled to 25 °C to obtain hydrogels.

### 2.5. Preparation of Bigels

Bigels were fabricated using the thermal emulsification technique [21]. Oleogels and hydrogels were weighed and mixed at oleogel-to-hydrogel mass ratios of 4:6, 5:5, 6:4, 7:3, and 8:2 (*w*/*w*), thoroughly stirred at 80 °C using a water bath magnetic stirrer to achieve melting and emulsification of both phases, and cooled to 25 °C to yield bigels. Bigels were stored in a refrigerator at 4 °C for 24 h.

### 2.6. Appearance Observation 

Bigels (15 mL) were transferred into 25 mL beakers for static incubation over 30 min to stabilize the system. The beakers were inverted and observed against a black background to obtain visual diagrams of apparent phase behavior, with photographs taken for documentation.

### 2.7. Microscopic Observation

Bigels were prepared on glass slides and gently pressed with coverslips. The observations of bigels were performed under 10× and 40× objective lenses using an optical microscope (BA200, Motic, Xiamen, China) with image acquisition conducted via a micro-imaging system.

Confocal laser scanning microscopy (CLSM) (TCS·SP8, Leica, Wetzlar, Germany) was performed according to a previously reported method, with slight modifications [22]. Bigels were stained with an ethanolic solution of Fluorescent Brightener 28 (1 mg/mL) for 5 min under light-shielded conditions, followed by another 5 min of staining with 10 μL of Nile red working solution. This dye was prepared by mixing 0.1 mg/mL and 0.9 mg/mL Nile red ethanol solutions at a volume ratio of 1:1 (*v*/*v*). Stained samples were spread on slides, covered, and observed. Excitation wavelengths were set to 552 nm (FITC), 488 nm (Nile red), and 405 nm (Fluorescent Brightener 28). The microstructures of bigels were observed under 10× and 40× magnifications with green fluorescence for protein signals, red fluorescence for oil-phase signals, and blue fluorescence for κ-carrageenan signals, respectively.

### 2.8. Fourier Transform Infrared Spectroscopy

Fourier transform infrared spectroscopy (FTIR) (Nicolet-iS50, Nicolet, Madison, WI, USA) was employed to characterize molecular structure and intermolecular interactions of bigels. Measurements were performed using ambient air as a blank reference, and an adequate volume of bigels was spread on the surface of the sample holder. Spectra were recorded between 4000 and 400 cm^−1^ with 32 scans at 25 °C.

### 2.9. Textural Properties

Texture profile analysis (TPA) of bigels was performed using a texture analyzer (TA-XT Plus, Stable Micro System, Godalming, UK) equipped with a *p*/0.5 cylindrical probe (diameter 12.7 mm). The trigger force was set to 5 g. The probe traveled a downward distance of 5 mm at a test speed of 1 mm/s, and the hardness, springiness, and cohesiveness of bigels were acquired.

### 2.10. Rheological Properties

The rheological properties of bigels were tested with minor adjustments to the experimental procedure reported in [23]. Bigels were positioned between the parallel plates of a rheometer (MCR-302, Anton Paar, Shanghai, China). A 25 mm diameter steel geometry was used, and the plate gap was adjusted to 1 mm at 25 °C.

Strain sweeps were run at 10 rad/s over 0.01–100% strain to locate the linear viscoelastic region. Frequency sweeps (0.1–100 rad/s) were run within the linear viscoelastic region, and flow curves (0.1–100 s^−1^) were recorded to monitor viscosity. For thixotropy measurement, a three-stage protocol was employed at 0.1 s^−1^ for 300 s, at 10 s^−1^ for 300 s, and at 0.1 s^−1^ for 300 s, respectively. For temperature sweep measurements, the angular frequency was fixed at 10 rad/s and the heating rate set to 5 °C/min. After coating silicone oil onto each bigel sample surface, the sample was positioned between parallel plates and heated from 10 °C to 100 °C, followed by cooling back to 10 °C. Creep recovery was assessed by applying 5 Pa for 300 s and then monitoring recovery for an additional 300 s. All experiments were repeated three times.

### 2.11. Statistical Analysis

All determinations were carried out with a minimum of three replicates, and acquired data were expressed as mean ± standard deviation. SPSS Statistics 25 and Origin 2022 were employed for one-way ANOVA, and differences were deemed statistically significant at *p* < 0.05.

## 3. Results

### 3.1. Appearance of Large Yellow Croaker MPs/κ-Carrageenan Bigels

**Figure 1:**
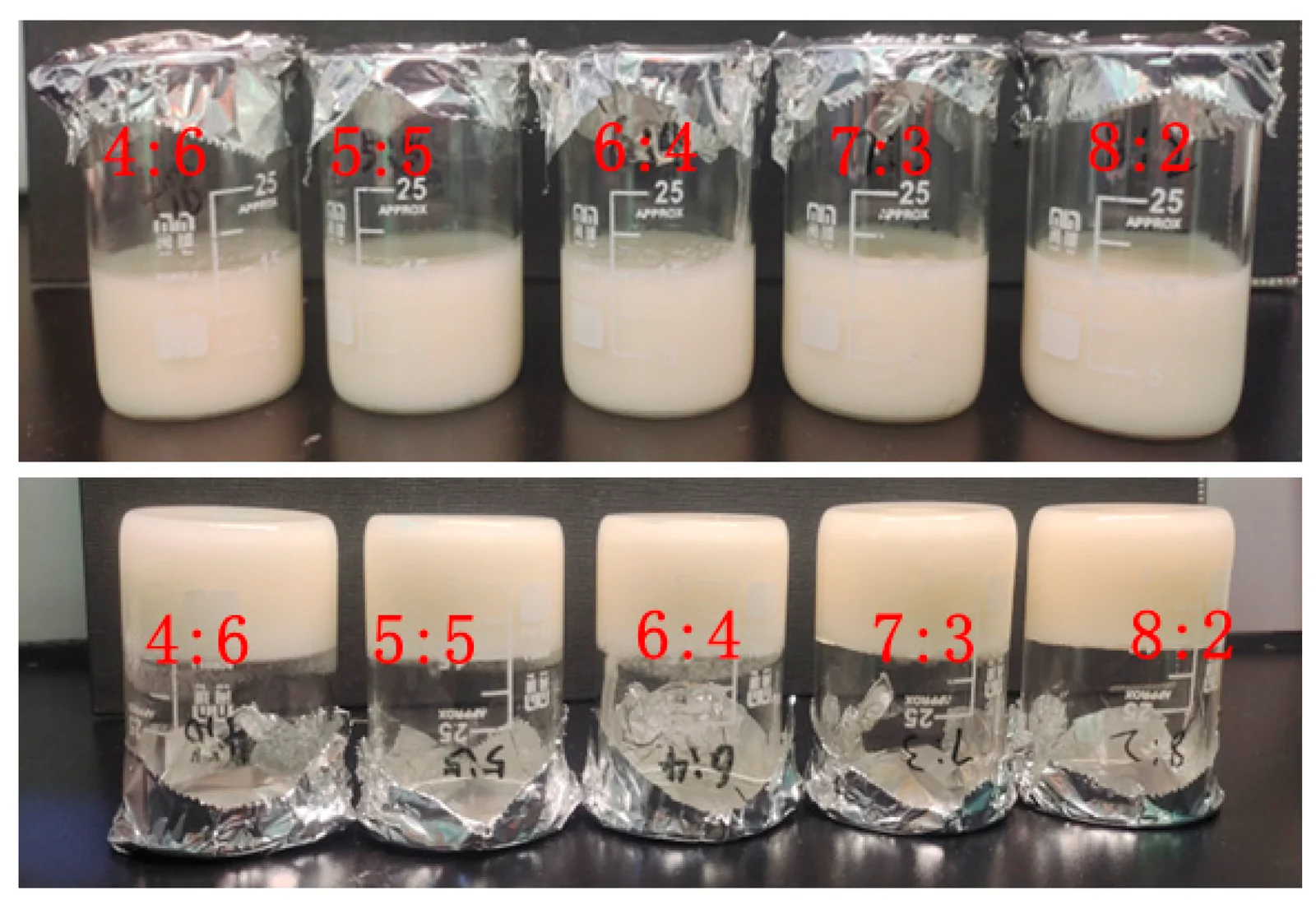
Appearance of large yellow croaker MPs/κ-carrageenan bigels at different oleogel-to-hydrogel ratios.

The appearance of large yellow croaker MPs/κ-carrageenan bigels as a function of oleogel-to-hydrogel volume ratio is presented in Figure 1. Bigels were arranged sequentially from left to right at oleogel-to-hydrogel ratios of 4:6, 5:5, 6:4, 7:3 and 8:2 (*w*/*w*), respectively. No flow behavior was observed after static inversion testing, and all resultant bigels presented a uniform milky-white appearance. This visual trait aligns with earlier records describing stable biphasic gels constructed from κ-carrageenan hydrogels and monoglyceride oleogels [8]. These results indicated that all the bigels possessed the solid-like self-supporting capability.

### 3.2. Microscopic Structure of Large Yellow Croaker MPs/κ-Carrageenan Bigels

**Figure 2:**
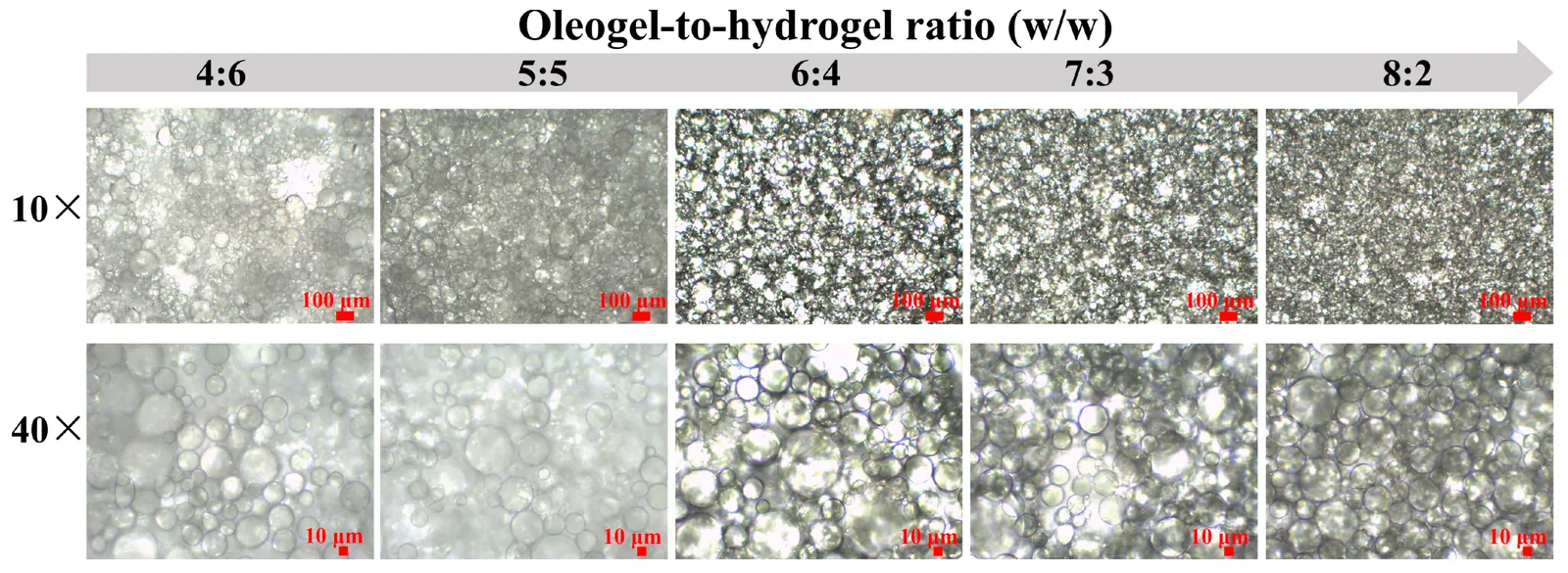
Optical micrographs of large yellow croaker MPs/κ-carrageenan bigels at different oleogel-to-hydrogel ratios.

Optical microscopy was adopted to characterize the microstructural morphology of bigels fabricated at diverse oleogel-hydrogel blending proportions, and the captured micrographs are displayed in Figure 2. As the proportion of oleogels increased, the droplets exhibited an increasingly pronounced tendency to adhere to each other. This trend corroborates earlier findings on bigels structured with κ-carrageenan hydrogels and various oleogelators (glycerol monostearate and beeswax) [24], indicating that the addition of oleogels altered the microstructure of bigels and enhanced interactions among different droplets. The structural evolution observed herein arises from intermolecular associations between oleogel and hydrogel phases, resulting in the rearrangement of the gel network and a modification in the compactness.

**Figure 3:**
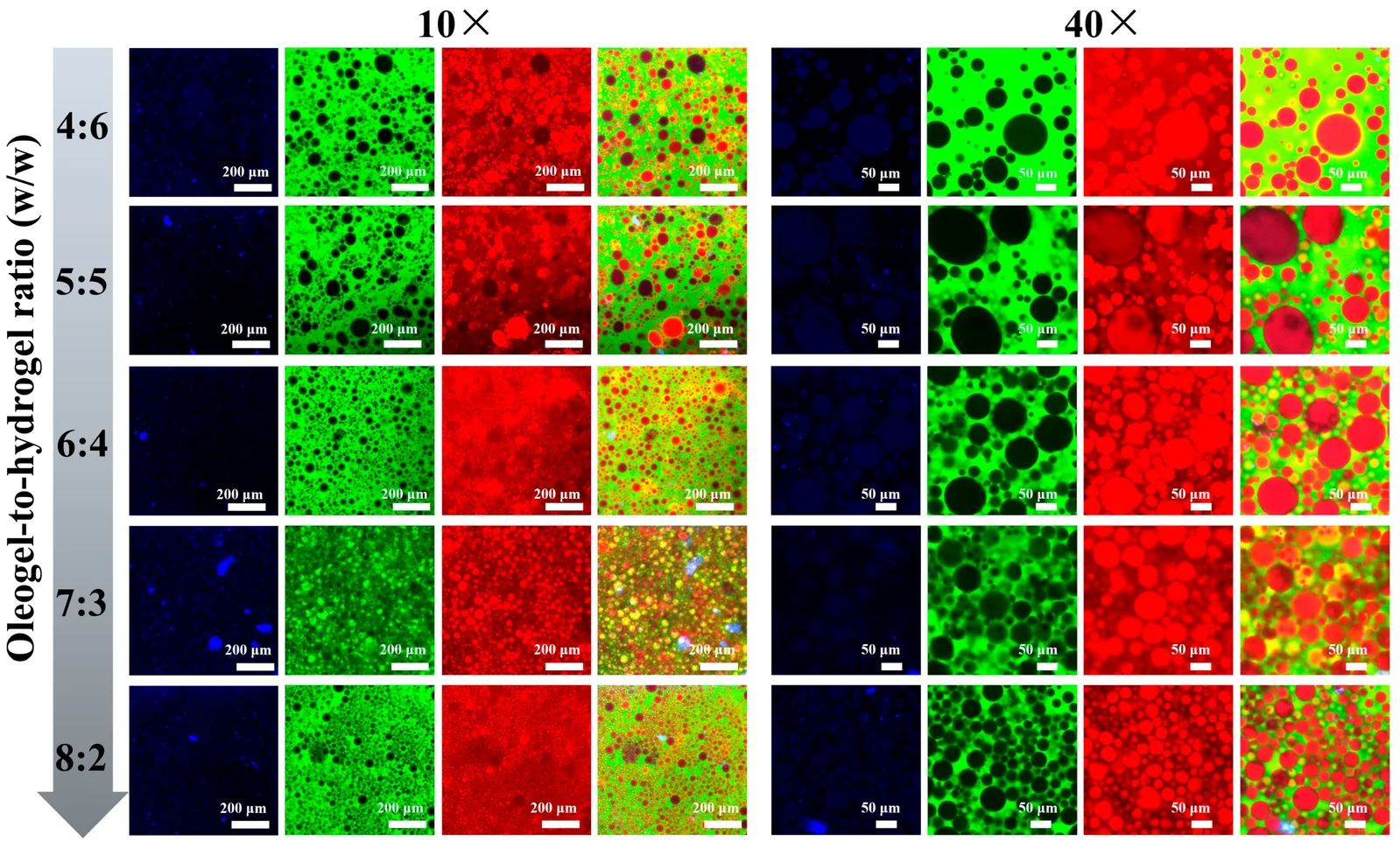
CLSM micrographs of large yellow croaker MPs/κ-carrageenan bigels with different oleogel-to-hydrogel ratios captured at various magnifications, where blue fluorescence represents the aqueous κ-carrageenan hydrogel phase, red fluorescence represents the oleogel phase, and green fluorescence represents protein components.

The distribution of hydrogels and oleogels in bigels was investigated by CLSM. As shown in Figure 3, the proportion of bright blue regions in merged images gradually decreased with increasing oleogel proportion. This phenomenon originated from changes in component ratios. As the oleogel fraction increased, the content of κ-carrageenan declined while soybean oil content rose, and the continuous aqueous domains were gradually occupied by dispersed oil phases. Bigels are categorized into O/W type, W/O type, and bicontinuous structures. Combined with fluorescence distribution features, the aqueous phase formed a continuous interconnected network in all formulations, and oleogels existed as independent spherical dispersed phases, preliminarily demonstrating that the obtained bigels belonged to the O/W type [25]. The ratio of oleogels to hydrogels is a key factor governing the type of bigels. Progressive elevation of the oleogel fraction narrows interdroplet gaps and boosts bigel viscoelasticity, yet dense droplet packing raises viscosity and hinders thermal homogenization, as observed in polysaccharide-based bigels [26]. Meanwhile, oleogel droplet size declines monotonically with higher oleogel ratios. Compact oil microspheres reduce inter-droplet collision, suppress coalescence/flocculation and avert macroscopic phase separation, improving long-term network stability; this mechanism agrees with prior reports on high-oil bigels [27].

### 3.3. FTIR Spectroscopy Analysis of Large Yellow Croaker MPs/κ-Carrageenan Bigels

**Figure 4:**
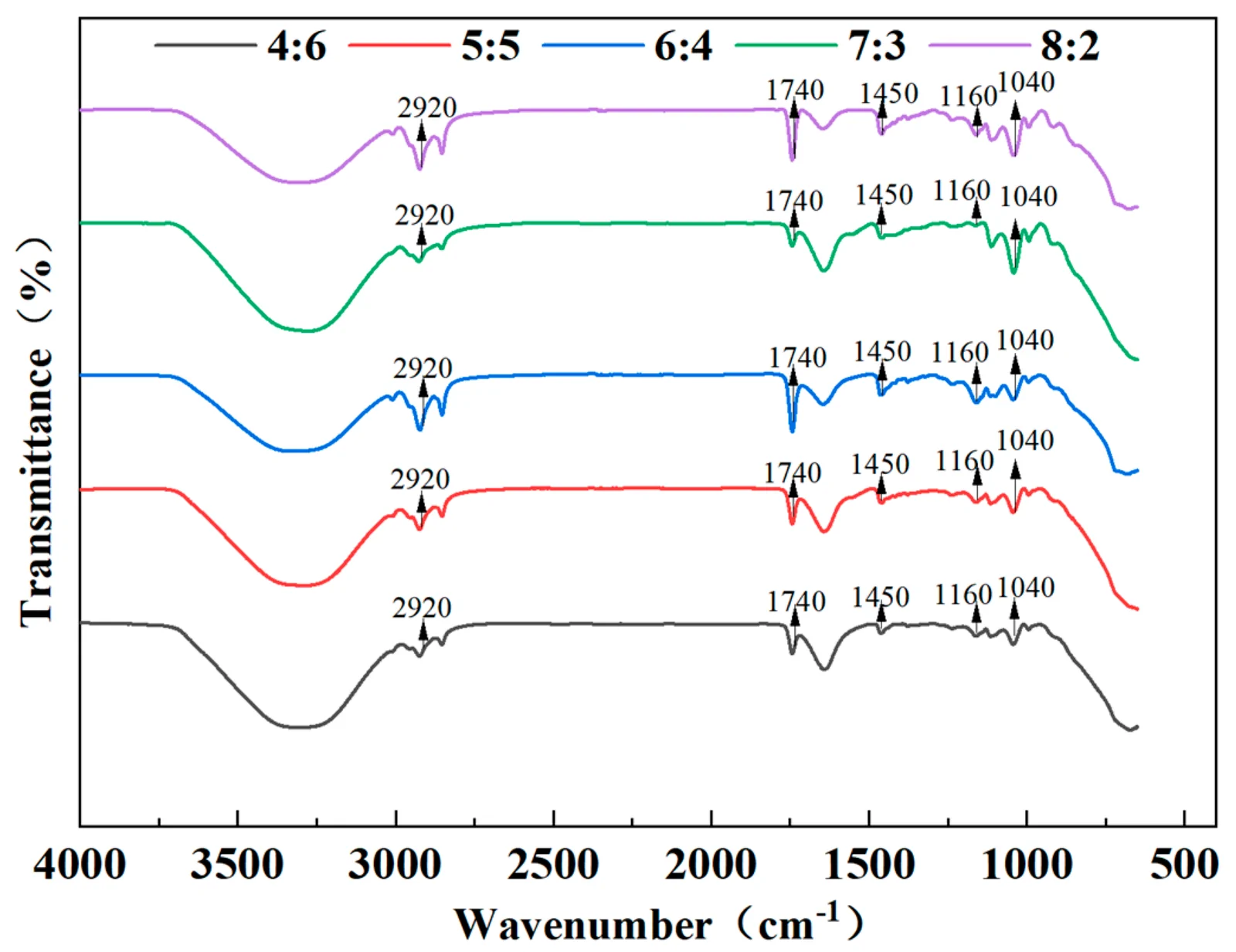
FTIR spectra of large yellow croaker MPs/κ-carrageenan bigels at different oleogel-to-hydrogel ratios.

FTIR serves as an important technology for characterizing molecular structures and elucidating intermolecular interactions. As shown in Figure 4, no new characteristic absorption bands or distinct peak shifts were observed across all samples. This finding merely suggests that substantial new covalent bonds were not generated during bigel fabrication, revealing that non-covalent intermolecular forces encompassing hydrogen bonds, hydrophobic aggregation, and electrostatic attractive forces serve as the primary interactions maintaining the intact network structure of the bigels [28]. Within the wavenumber scope of 3000–3600 cm^−1^ lies a broad absorption signal, corresponding to the stretching motion of O–H bonds. This peak likely originated from hydrogen-bonded hydroxyl groups present in both κ-carrageenan and the oleogel components. As the fraction of oleogel components rises, the intensity of characteristic absorption peaks declines gradually, revealing the weakened relative effect of hydrogen-bonding interactions inside the system [25]. The successive sharp absorption peaks near 2920 cm^−1^ were assigned to the C–H stretching vibrations of triglycerides and monoglycerides. The absorption band at 1450 cm^−1^ corresponded to C–H stretching vibrations, confirming the presence of saturated and unsaturated fatty acyl chains originating from soybean oil in the bigels. The characteristic peaks at 1744 cm^−1^ and 1160 cm^−1^ were attributed to C=O stretching vibrations, whereas the signal at 1638 cm^−1^ arose from C–H bending vibrations. The absorption bands at 1160 cm^−1^ and 1040 cm^−1^ were assigned to the C–O stretching vibrations of C–O–H and glycosidic C–O–C groups in κ-carrageenan molecules, respectively [29]. These results were similar to those previously reported about edible bigels fabricated via blending starch-based hydrogels and monoglyceride-rich oleogel matrices, indicating that the network structure of bigels was partially maintained by both hydrogen bonds and covalent Schiff base linkages [30] .

### 3.4. Texture Analysis of Large Yellow Croaker MPs/κ-Carrageenan Bigels

From the data in Table 1, the oleogel addition ratio significantly modulated the mechanical performance of bigels. Hardness displayed a tendency of first increasing and then decreasing with elevated oleogel proportion, achieving the peak value of 11.67 ± 0.56 g at an oleogel-to-hydrogel ratio of 5:5 (*w*/*w*), which was significantly higher than that of all other ratios (*p* < 0.05). The incorporation of oleogels disrupted the hydrogel network structure, leading to decreased cohesiveness, enhanced fluidity, and reduced shear resistance of bigels, consistent with previous reports [31]. This phenomenon arises from dilution of the hydroxyl-rich aqueous matrix by elevated oil fractions, which reduces hydrogen bond density between large yellow croaker myofibrillar proteins and κ-carrageenan and weakens the overall crosslinking strength of the bigel network [32]. Once the oleogel fraction exceeded the critical threshold, the textural properties of bigels deteriorated remarkably. Textural parameters including elasticity, chewiness, and springiness showed changing trends consistent with hardness.

### 3.5. Rheological Properties of Large Yellow Croaker MPs/κ-Carrageenan Bigels

**Figure 5:**
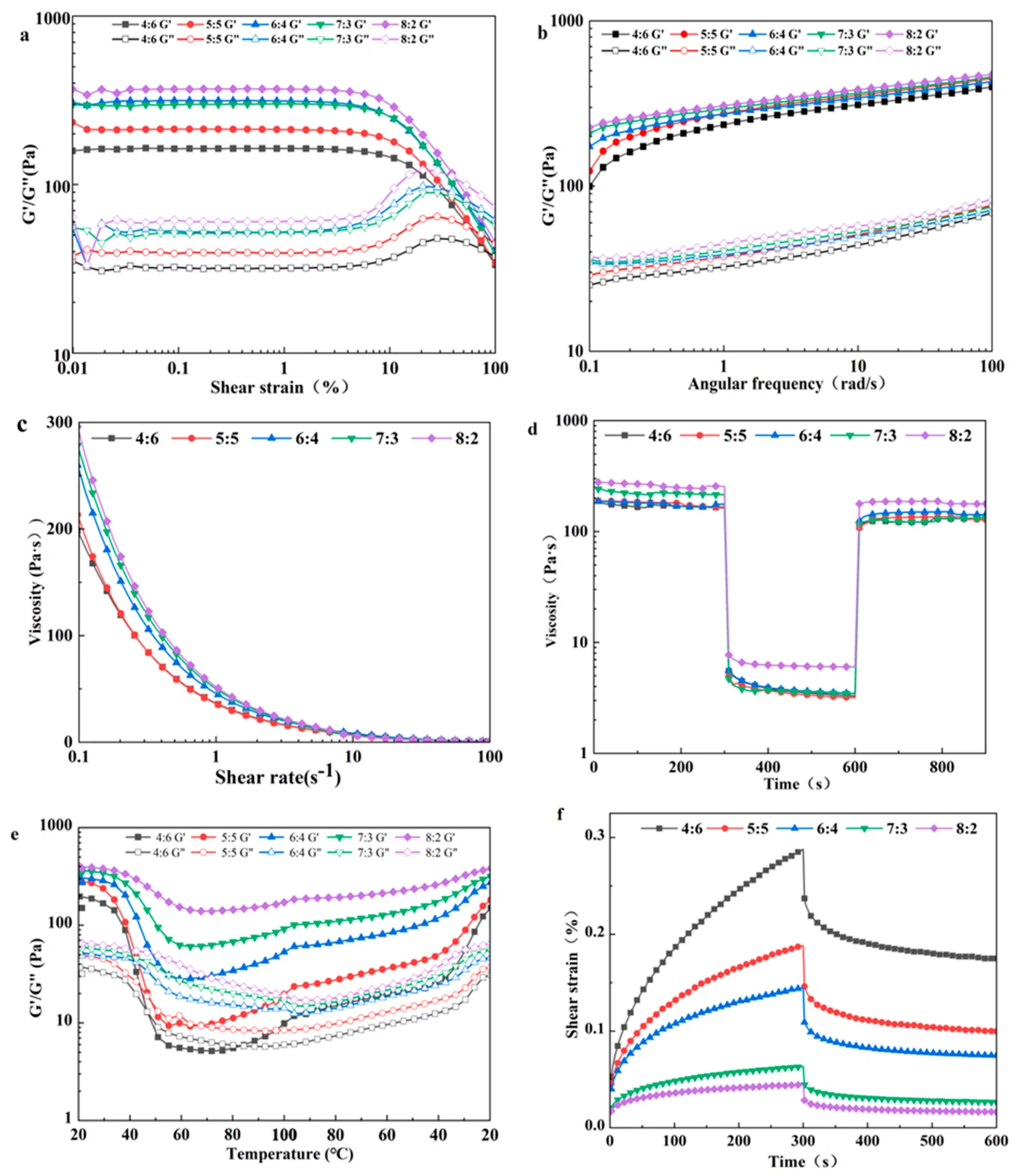
Rheological properties of large yellow croaker MPs/κ-carrageenan bigels with different oleogel-to-hydrogel ratios in (**a**) strain sweep, (**b**) frequency sweep, (**c**) apparent viscosity, (**d**) thixotropic curve, (**e**) temperature sweep, and (**f**) creep curve.

Strain sweep serves to define the linear viscoelastic domain by adjusting shear strain magnitudes and to analyze the viscoelastic properties of bigels by comparing the variation rules and mutual relationship of G′ and G″. As shown in Figure 5a, all the bigels exhibited G′ > G″ over a certain range, demonstrating that elastic properties dominated within the linear viscoelastic regime [10]. In the linear viscoelastic zone, G′ and G″ showed a synchronous upward trend with growing oleogel ratios. Meanwhile, bigel architectures possessed comparatively steady structures when subjected to shear force. This behavior resembles that of an elastic solid, which can store the energy applied during shearing. When the shear strain exceeded the linear viscoelastic region, bigels exhibited nonlinear behavior, due to a decrease in G’ and G″ with increasing shear strain. When G′ equaled G″, it was referred to as the critical yield point. At this point, the structures of bigels collapsed, and their elastic behavior transitioned to plastic behavior [33]. G’ in the strain sweep curves of all the bigels exhibited a decrease to indicate the presence of shear yield behavior in bigels.

Figure 5b reveals that both G′ and G″ rose proportionally with increasing oleogel fraction, suggesting that the oleogel-to-hydrogel ratio directly modulates bigel viscoelasticity. Higher oleogel contents led to denser packing of the dispersed phase, reinforcing the network structure and thus elevating G′ [34]. Bigels at all the proportions exhibited G′ > G″ to demonstrate that these bigels possessed solid-like gel characteristics.

Flow curves were established based on the apparent viscosity data of bigels obtained at various shear rates to judge their flow properties and storage stability [30]. Figure 5c shows a positive correlation between apparent viscosity and oleogel fraction, with viscosity rising progressively. The bigels displayed shear-thinning flow characteristics when shear rate grew, consistent with previous studies concerning bigel systems constructed from potato starch hydrogel and glycerol monostearate oleogel [32]. The internal network structure of bigels gradually unfolded under the action of shear force, thereby enhancing the fluidity of the bigels [35]. This observation may be ascribed to the fact that shear action disrupted the internal structure of the bigels as well as the interactions between hydrogels and oleogels.

The three-interval thixotropy test was employed to evaluate the structural dissociation and recovery of bigels under alternating shear [33]. As shown in Figure 5d, all bigels exhibited sharply reduced viscosity under high shear (>10 s^−1^), which was lower than the viscosity measured in the initial low-shear stage. Varying degrees of viscosity recovery were observed at low shear rates in the recovery stage, demonstrating that bigel networks possessed structural recoverability. Elevated oleogel fractions brought about a synchronous rise in the baseline viscosity of composite bigels. The thixotropic recovery index was then determined as the ratio of the initial apparent viscosity from the third cycle to the final apparent viscosity from the first cycle. The thixotropic recovery of the bigels at ratios of 4:6, 5:5, 6:4, 7:3, and 8:2 (*w*/*w*) was 67.42%, 64.67%, 71.07%, 54.14%, and 69.76%, respectively. Notably, pork MP gels show no thixotropic recovery under shear [36], whereas the present bigels exhibit a distinct three-stage response with 71.07% recovery, owing to the high flexibility of fish myosin tails that facilitates rapid reformation of non-covalent networks upon shear cessation. The ratio-dependent recovery reflects a balance between network disruption and reformation, with partial irreversible breakdown occurring in all formulations within the test period [30].

As shown in Figure 5e, during the early heating stage (10–30 °C), the modulus of all the bigels remained almost constant. When the temperature rose to the critical point (40 °C), the G’ of bigels began to decrease rapidly, indicating the instability of bigels. The G’ of bigels exhibited the most rapid decrease within the temperature range of 40–60 °C, indicating that the structure of the bigels underwent disintegration, which was attributed to the breakdown of oleogels and hydrogels. The G″ > G’ for most of the bigels at temperatures above 60 °C, which indicated that the bigels had transformed into a liquid state [37]. The above change arises owing to the thermoreversibility of oleogel and hydrogel phases in the bigels. During the cooling process, the storage modulus G′ of bigels rose moderately as the system temperature dropped. This demonstrated that the temperature-induced evolutionary pattern of bigels’ rheological performances was strongly linked to the blending ratio of oleogels and hydrogels. Notably, the 40 °C structural transition matches the 40–50 °C denaturation range of fish myosin, indicating protein network breakdown dominates thermal collapse [38]. By contrast, terrestrial myosin denatures at 55–58 °C [39]. Owing to this low-temperature response, marine protein bigels are more suitable for thermolabile bioactive encapsulation and cold processing than terrestrial protein counterparts.

Creep tests were performed to assess the creep-recovery response of bigels under a constant shear stress. As shown in Figure 5f, the critical shear strain of bigels fabricated from large yellow croaker MPs and κ-carrageenan declined gradually with the rising fraction of oleogels. The bigels exhibited pronounced viscous flow characteristics in the initial stage at a shear stress of 5 Pa from 0 to 300 s, indicating that the bigels had undergone deformation and even potential flow behavior. As the loading time elapsed, the bigels exhibited distinct elastic response characteristics under the action of sustained shear stress. When the external shear stress was instantaneously reduced to zero, the bigel systems at 300 s were in a stretched state to resist the applied stress, and initiated their relaxation mechanism and entered the deformation recovery phase. In the stress-free recovery phase from 300 s to 600 s, the bigels attempted to recover their original morphology, with the strain values dropping rapidly. The bigels continued to undergo recovery through viscoelastic relaxation as time progressed to reflect the balance of viscoelastic properties within the bigels.

## 4. Conclusions

In this study, bigels were fabricated from large yellow croaker myofibrillar protein-soybean oil oleogels and κ-carrageenan hydrogels; the resultant biphasic gels exhibited solid-like self-supporting properties and stable internal structures. Texture analysis confirmed that oleogel content significantly influenced the textural firmness of the bigels (*p* < 0.05). Furthermore, excessive oleogel proportion disrupted the hydrogel crosslinked network and weakened the overall mechanical performance of bigels. Rheological properties showed that the bigels exhibited solid-like gel behavior at low strain (G’ > G″), while the structure was destroyed at high strain. Apparent viscosity increased progressively with oleogel proportion, and all bigels displayed shear-thinning non-Newtonian flow behavior. The G’ of bigels decreased rapidly above 40 °C, and G″ was higher than G’ above 60 °C. These experimental findings offer important references for developing innovative raw materials for healthy foods. Collectively, stable bigels were fabricated by regulating the volume ratio of oleogel to hydrogel phases, using large yellow croaker MP–soybean oil oleogels and κ-carrageenan hydrogels as precursors. Future research will further explore the stability in different environments. This study provides feasible strategies for the manufacture of marine protein-based fat replacers, and promotes high-value valorization of large yellow croaker processing by-products as well as broader application of such biphasic gels in the healthy food sector.

## Figures and Tables

**Table 1:** Textural properties of large yellow croaker MPs/κ-carrageenan bigels at different oleogel-to-hydrogel ratios.

Oleogel-to-Hydrogel Ratios	Hardness/g	Elasticity	Cohesiveness	Chewiness	Springiness
4:6	9.73 ± 0.64 ^b^	0.99 ± 0.01 ^a^	0.73 ± 0.01 ^a^	7.35 ± 0.3 ^b^	0.29 ± 0.03 ^a^
5:5	11.67 ± 0.56 ^a^	0.97 ± 0.01 ^b^	0.73 ± 0.03 ^a^	8.32 ± 0.83 ^a^	0.3 ± 0.02 ^a^
6:4	8.95 ± 0.91 ^b^	0.96 ± 0.01 ^bc^	0.69 ± 0.03 ^a^	5.8 ± 0.57 ^c^	0.2 ± 0.01 ^b^
7:3	7.05 ± 0.05 ^c^	0.96 ± 0 ^bc^	0.71 ± 0.02 ^a^	4.8 ± 0.16 ^d^	0.17 ± 0.01 ^b^
8:2	6.05 ± 0.13 ^c^	0.96 ± 0.01 ^c^	0.7 ± 0.02 ^a^	4.05 ± 0.18 ^d^	0.12 ± 0.01 ^c^

Values are expressed as mean ± SD (*n* = 5), and distinct superscript marks within an identical column denote statistical disparities (*p* < 0.05).

## Data Availability

The original contributions presented in the study are included in the article, further inquiries can be directed to the corresponding author.
